# Successful treatment of pityriasis lichenoides et varioliformis acuta with stapokibart: a case report

**DOI:** 10.3389/fmed.2025.1615108

**Published:** 2025-09-01

**Authors:** Rongqiao Shi, Aijuan He, Zunfeng Ma, Mengying Jiang

**Affiliations:** ^1^Guizhou University of Traditional Chinese Medicine, Guiyang, China; ^2^Department of Dermatology, First Affiliated Hospital of Guizhou University of Traditional Chinese Medicine, Guiyang, China

**Keywords:** stapokibart, pityriasis lichenoides et varioliformis acuta, inflammatory skin disorder, immunomodulation, case report

## Abstract

Pityriasis lichenoides et varioliformis acuta (PLEVA) is a relatively rare inflammatory skin disorder encountered in clinical practice. Stapokibart is a humanized monoclonal antibody drug targeting the interleukin receptor subunit α (IL-4Rα), and is primarily used for the treatment of atopic dermatitis. Currently, owing to the rarity of PLEVA in clinical practice, there have been no reports on the use of Stapokibart in its treatment. This article presents a case in which stapokibart was used to treat a patient with acute acneiform lichenoid pityriasis, demonstrating its clinical efficacy. Following treatment with biological agents, the patient’s rash completely resolved, and their quality of life significantly improved, without any adverse reactions. This case provides a valuable reference for the clinical treatment of PLEVA.

## 1 Introduction

Pityriasis lichenoides et varioliformis acuta (PLEVA) is a rare inflammatory skin disorder with an unclear etiology and no established standard treatment. Stapokibart inhibits the release of inflammatory factors by blocking the binding of interleukin (IL-4) and IL-13 to interleukin receptor subunit α (IL-4Rα). This article describes the case of a patient with PLEVA who was successfully treated with stapokibart. The patient was initially injected with a dose of 600 mg, followed by 300 mg subcutaneously every 2 weeks. After 2 weeks of treatment, pruritus was significantly reduced, and the rash began to resolve after 6 weeks. Complete resolution of the rashes was observed after 12 weeks, showing the effectiveness of the treatment. No recurrence was noted during a 3-month follow-up period. Therefore, this case report highlights the clinical efficacy of stapokibart in the treatment of PLEVA.

## 2 Case description

**FIGURE 1:**
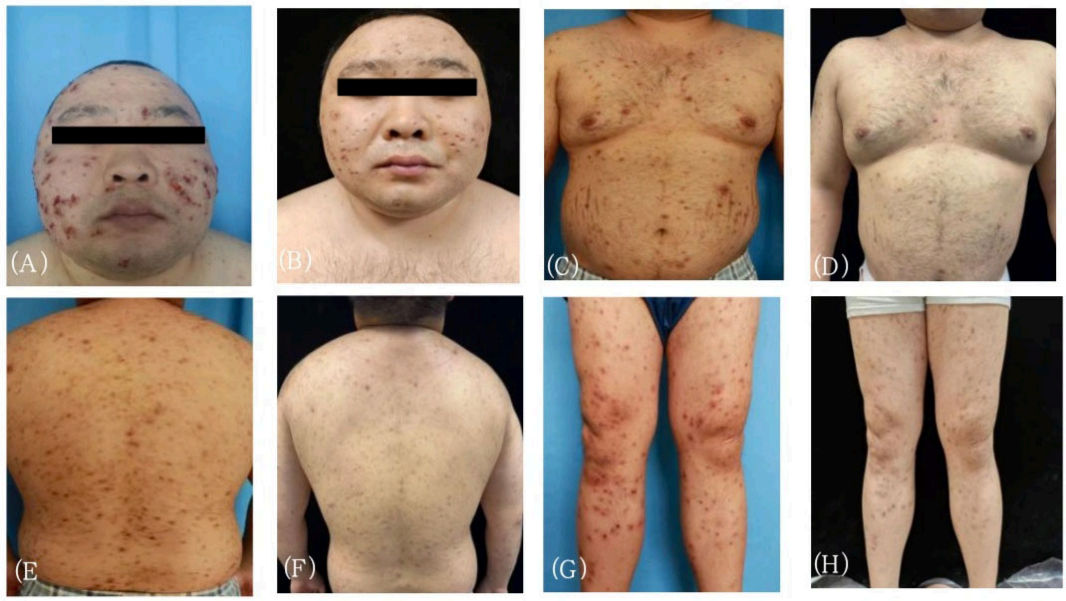
Observe the condition of skin lesions before and after treatment with stapokibart. **(A,C,E,G)** Before treatment. **(B,D,F,H)** Week 12 of treatment.

**FIGURE 2:**
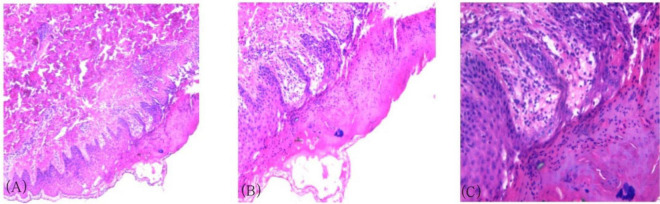
Histopathological results of the skin on the left arm. **(A)** Hyperkeratosis of the epidermis, inflammatory cell aggregation in the stratum corneum, localized acanthosis in the stratum spinosum (HE × 40). **(B)** Liquefactive degeneration of basal layer cells, superficial dermal interstitial edema (HE × 100). **(C)** Perivascular lymphocytic infiltration with endothelial cell swelling (HE × 200).

A 27-year-old male patient presented with erythema, papules, vesicles, and pruritus, with recurrent episodes lasting more than 2 years. Approximately 2 years ago, he developed scattered papules, nodules, and vesicles on his body without any obvious triggers. These lesions were accompanied by severe itching that affected his daily activities and sleep. Scratching led to vesicle rupture and crust formation. Over time, the rash spread throughout the body, leading to hospitalization at several medical facilities, Following a skin biopsy, PLEVA was diagnosed. Initial treatment with tofacitinib, thalidomide tablets, compound glycyrrhizin tablets, methotrexate, ultraviolet phototherapy, and other treatments, resulted in mild improvement. One year ago, the patient experienced a recurrence of symptoms, again without any obvious trigger. The rash worsened, with marked pruritus and pain. He was admitted to our department, and a second skin biopsy confirmed the diagnosis of PLEVA. Treatment with immunoglobulin, methylprednisolone, sodium succinate, anti-infection therapy, and immunomodulation led to clinical improvement. After discharge, the patient continued regular oral treatment with oral methylprednisolone tablets, cyclosporine capsules, and Tripterygium wilfordii tablets. His symptoms initially improved with these treatments; however, the rash repeatedly recurred. Four days before his most recent hospitalization, the patient experienced symptom exacerbation following a common cold. This episode was characterized by the appearance of numerous papules, nodules, and vesicles on the neck, chest, abdomen, and limbs, with significant itching, along with headache and nasal congestion. The patient had a history of atopic dermatitis. Systemic examination showed stable vital signs. There was a cushing syndrome due to the patient’s prolonged corticosteroid use; however, no other abnormalities were found. Dermatological examination revealed numerous scattered, round, red patches and papules ranging in size from mung bean- to soybean-sized on the head, face, trunk, and limbs, along with a few scattered blisters. The blister walls were tense, the blister fluid was clear, and some lesions ruptured, with crusting and blood scabs. The body also had numerous scattered point- and line-shaped scratch marks. No obvious pustules or hyperplasia were observed. Scattered hyperpigmentation and acne-like scarring were observed after healing (Figures 1A, C, E, G). Laboratory test results were as follows: White blood cell count, 18.81 × 10^9^/L; lymphocyte percentage, 8.60%; eosinophil percentage, 15.70%; absolute neutrophil count, 13.30 × 10^9^/L; and absolute eosinophil count, 2.95 × 10^9^/L. Allergen testing indicated a positive reaction to house dust mites, egg white, and wheat. A total immunoglobulin E (IgE) level of 97,500 IU/mL was found 2 years ago; at admission to our department, the level exceeded 1000 IU/mL and was retested after 2 months of using stapokibart. Serological testing was positive for Epstein-Barr virus (EBV) and varicella-zoster virus (VZV) antibody IgG. Regarding the lymphocyte count, the natural killer cell (CD16 + 56) count was 79.97 cells/μL, and the B lymphocyte percentage (CD19) was 19.17%. Kidney function testing indicated a uric acid level of 502 μmol/L. Liver function tests showed no significant abnormalities. Skin histopathology of the left arm showed hyperkeratosis of the epidermis, with infiltration of inflammatory cells in the stratum corneum, localized thickening of the stratum spinosum, liquefaction degeneration of the basal layer cells, superficial dermal edema, and perivascular infiltration with endothelial cell swelling (Figures 2A–C). Based on the patient’s clinical presentation and pathological results, the patient was diagnosed with PLEVA.

## 3 Therapeutic evaluation

After the patient was admitted to the hospital, he was initially treated with cyclosporine capsules, roxithromycin dispersible tablets. However, the effect was unsatisfactory after topical relapsing polymyxin B ointment. After communicating with the patient, consent was obtained for systemic administration of steroids. The patient was administered 40 mg of intravenous methylprednisolone sodium succinate as an anti-inflammatory therapy. This resulted in the control of the facial rash; however, the patient still experienced pain. Therefore, stapokibart therapy was recommended. After excluding contraindications and obtaining consent from both the patient and their family, stapokibart therapy was initiated. The first dose was 600 mg, with subsequent doses of 300 mg every 2 weeks. The patient was instructed to receive subcutaneous injection of 300 mg of stapokibart and 30 mg of oral prednisone acetate every 2 weeks, with the glucocorticoid dosage adjusted at regular outpatient visits. After 3 months of treatment, a total of eight subcutaneous injections of stapokibart were given, and the glucocorticoid dosage was reduced to 15 mg.

After 2 weeks of treatment with stapokibart, the patient had dry lesion areas on the trunk and extremities, depressed scarring, a few scattered scabs on the head, face, and buttocks, and no pustules in the lesion areas, with a visual analog scale (VAS) score of 4, and a dermatology Life Quality Index (DLQI) score of 10. After the 6th week, the patient’s condition improved, with no new blisters, and scattered superficial scarring and brown hyperpigmented spots were observed. The VAS score had decreased to 3,and the DLQI score to 6. After the 10th week, the patient’s rash improved significantly, with scattered hyperpigmentation and acne-like scarring, and the VAS score was 2,and the DLQI score to 3. After the 12th week, the original skin lesions subsided, leaving acne-like scarring (Figures 1B, D, F, H), and the VAS score was 1,and the DLQI score to 2. After 3 months of follow-up, no new skin lesions were observed. The follow-up kidney function results indicate a uric acid level of 473 μmol/L. In terms of safety, stapokibart was well tolerated by the patient and no serious adverse reactions or other conditions were observed, indicating a high-level safety profile.

## 4 Discussion

PLEVA is a self-limiting disease with an acute onset and variable duration, which is prevalent in young and middle-aged males between the ages of 20 and 30 years. It is typically characterized by scaly erythematous plaques, papules, papules, blisters, necrotic, and crusted polymorphous damage, with hyperpigmentation and pimple-like scarring remaining after the scabs have fallen off (1). The condition was first reported by Degos et al. (2), and only a few cases have been reported both domestically and internationally. The pathogenesis of PLEVA remains unclear, although it is primarily associated with infection, abnormal T-cell proliferation, and immune complex-mediated hypersensitivity. The pathogens found to be associated with the pathogenesis of PLEVA include varicella zoster virus, EBV, and microviruses (3). PLEVA is relatively rare, and no standardized treatment regimen exists, and the efficacy of therapeutic outcomes vary depending on the age at disease onset and disease duration. Combination regimens can be used, and the recommended first-line treatment regimen includes oral antibiotics, topical glucocorticoids, or immunomodulators. The second-line regimens included UVB or PUVA. The third-line therapeutic agents include methotrexate, amiloride, and cyclosporine (4). Currently, glucocorticosteroids are the first-line treatment for PLEVA; however, achieving clinical remission requires a prolonged tapering period, during which patients not only face the possibility of relapse but also experience the adverse effects associated with long-term glucocorticosteroid use. Therefore, after excluding contraindications to the use of medication and obtaining the consent of the patients and their families, we considered the use of a combination of biologics.

On September 12, 2024, the State Drug Administration (SDA) approved stapokibart monotherapy injection for use in the treatment of moderate-to-severe atopic dermatitis in adults whose disease is poorly controlled by, or not suitable for, topical medications (5). Subsequently, stapokibart additionally received approval in China for use in the treatment of chronic rhinosinusitis with nasal polyps (December 2024) and for the treatment of seasonal allergic rhinitis (February 2025) (6). Stapokibart is a novel humanized monoclonal antibody drug used to treat diseases caused by excessive inflammation. It targets IL-4Rα by preventing IL-4 and IL-13 from binding to IL-4Rα, and by inhibiting downstream inflammatory factor release, protein expression, and inflammatory cellular activity induced by IL-4 and IL-13 (5). Stapokibart has unique epitopes and differential cross-species responsiveness compared with dupilumab (6). Studies have shown that stapokibart exhibits blocking activity comparable to that of dupilumab, effectively inhibiting the binding of IL-4 and IL-13/IL-13Rα-1 complex to IL-4Rα. *In vitro* experiments have confirmed that its biological activity is comparable to or slightly superior to that of dupilumab (7).

Given that the patient in the present case presented with significantly elevated IgE and eosinophil levels, along with a history of atopic dermatitis, we speculated that PLEVA was associated with a type 2 inflammation mechanism. There are few reports on the use of biologics in PLEVA treatment, and previous literature has described the successful treatment of PLEVA with dupilumab, where after 3 weeks, the patient’s lesions almost completely disappeared, serum IgE levels significantly decreased, and there was no relapse after a 3-month follow-up. It was considered that PLEVA was associated with a type 2 inflammation mechanism, driven by the T-helper 2 cytokines, particularly IL-4 and IL-13 (8). This also provides support that the development of PLEVA is closely related to type 2 inflammation.

Currently, no previous studies have reported on the use of stapokibart to treat PLEVA, and the specific roles of type 2 inflammation, IL-4/13, and IgE in its pathogenesis remain unclear. We will further explore the pathogenesis of PLEVA and the role of type 2 inflammation in the course of efficacy observations.

In previous studies, T cell infiltration has been observed in patients with PLEVA, and CD8 + T cells were predominant (9). CD8 + T cells can be categorized into the Tc1 and Tc2 subgroups, with Tc2 cells secreting IL-4, IL-5, and IL-13 (10). Additionally, some cases have shown IL-13 expression in both CD4 + and CD8 + T cells (11). Therefore, stapokibart may act by attenuating the increase in IL-4 and IL-13 levels associated with Tc2, which is induced by T cell proliferative diseases. The patient showed significant improvement after 2 weeks of stapokibart treatment, and after 12 weeks of regular treatment, no relapse was observed.

## 5 Conclusion

This case report describes the successful treatment of PLEVA using stapokibart. The patient had a disease duration of over 2 years, with long-standing symptoms of generalized rashes and itching, accompanied by elevated eosinophil and IgE levels, and a history of atopic dermatitis. Symptoms did not improve with conventional treatment. Given that stapokibart effectively suppressed type 2 inflammation, targeted therapy with stapokibart was administered. After 2 weeks of biological treatment, the patient’s skin lesions showed significant improvement. During the 3-month follow-up period, glucocorticoids were reduced from 30 mg to 15 mg, and no disease recurrence or adverse events were observed, indicating good control of the patient’s condition. The evaluation of regular kidney function indicated a good safety profile. According to a review of relevant domestic and international literature, this is the first case of severe PLEVA treated with stapokibart. However, given that this study included only one patient and the observation period was short, further clinical research is needed to verify the efficacy and safety of stapokibart in the treatment of PLEVA. Additionally, further research and observations are needed to determine whether other patients with PLEVA could benefit from stapokibart.

## Data Availability

The original contributions presented in this study are included in this article/supplementary material, further inquiries can be directed to the corresponding author.
